# Research progress on developmental characteristics and clinical intervention of restricted and repetitive interests/behaviors in autism spectrum disorder across the lifespan: a narrative review

**DOI:** 10.3389/fpsyt.2026.1995599

**Published:** 2026-10-30

**Authors:** Lei Zhou, Ying Dai

**Affiliations:** 1 Growth, Development and Mental Health Center of Children and Adolescents, Children’s Hospital of Chongqing Medical University, Chongqing, China; 2 Chongqing Key Laboratory of Child Neurodevelopment and Cognitive Disorders, Chongqing, China; 3 National Clinical Research Center for Children and Adolescents’ Health and Diseases, Chongqing, China; 4 Ministry of Education Key Laboratory of Child Development and Disorders, Chongqing, China

**Keywords:** across the lifespan, autism spectrum disorder, insistence on sameness, repetitive sensorimotor, restricted and repetitive interests/behaviors

## Abstract

Restricted and repetitive interests/behaviors (RRBs) are a core symptom domain of autism spectrum disorder (ASD), with a significant impact on social interaction, learning, and overall functioning of autistic individuals. Clinical features of RRBs in ASD encompass repetitive sensorimotor (RSM) behaviors and insistence on sameness (IS). Taking a lifespan approach, this narrative review synthesizes current understanding of the neurobiological and psychological mechanisms associated with RRBs, together with their clinical manifestations during each developmental phase, and discusses recent advances in clinical interventions. Importantly, the available evidence is not distributed evenly across the lifespan: RRBs have been studied far more extensively in early childhood, whereas evidence concerning middle-aged and older autistic adults remains very limited. This review therefore does not imply that the evidence base is equally strong at every life stage. It aims to provide a practical clinical reference for professionals in pediatrics, rehabilitation medicine, and related fields.

## Introduction

Autism spectrum disorder (ASD) is a neurodevelopmental disorder distinguished by difficulties in social interaction, together with the manifestation of restricted, repetitive behaviors or interests (RRBs) (1). Termed a “spectrum” disorder, ASD encompasses a wide variety of clinical manifestations, exhibiting significant heterogeneity in the severity of symptoms, cognitive and linguistic abilities, and the existence of concomitant conditions (2). In recent decades, there has been a notable increase in the prevalence of ASD, with current estimates suggesting it affects approximately 3 in 100 children in America (3). Prevalence estimates vary across regions and are influenced by methodological differences in case ascertainment, which complicates direct international comparison (4). This elevation is likely a result of various factors, including shifts in diagnostic criteria, advancements in screening and diagnostic tools, as well as increased awareness among the public and healthcare practitioners (5).

Encompassing a broad spectrum of behaviors, the RRB domain is fundamental to ASD diagnosis, including stereotyped motor movements, repetitive utilization of items, the strong emphasis on uniformity, highly restricted interests, and atypical sensory responses (6). These behaviors go beyond being diagnostic markers; they commonly pose a significant hindrance to learning, hinder the acquisition of social skills, and can lead to considerable stress for both the individuals and their families (6). Despite their clinical importance, research and intervention resources for ASD have long been predominantly focused on the social communication domain, with relatively insufficient exploration into the causes, developmental patterns, and intervention strategies for RRBs (6, 7).

With progress in research methods in recent years, the scholarly understanding of RRBs is experiencing significant shifts. A particularly significant shift is the reevaluation by researchers of the importance and specificity of RRBs in the diagnosis of ASD. The traditional view asserts that social impairment is the fundamental characteristic of ASD; however, in clinical practice, many other neurodevelopmental disorders or mental conditions may also manifest social difficulties. Therefore, accurately distinguishing individuals with true ASD from the referred population with prevalent social issues poses a significant challenge. An innovative study utilizing large-scale language models (LLMs) deconstructed the diagnostic logic of experts in thousands of clinical reports. The results revealed that stereotyped repetitive behavior (DSM-5 criterion B1), special interests (B3), and sensory-based behaviors (B4) showed more significance and predictive power in distinguishing confirmed ASD from cases initially suspected but later ruled out than social interaction deficits (Criterion A) (8).

In recent years, an expanding body of research has started to elucidate the intricacies of RRBs, demonstrating their non-monolithic nature. Various studies have constantly recognized specific subtypes of RRBs, typically classified under a two-factor model: “Repetitive Sensorimotor” (RSM) behaviors and “Insistence on Sameness” (IS) behaviors (9). “Repetitive Sensorimotor” (RSM) behaviors, often categorized as “lower-level,” include actions such as flapping one’s hands, finger-wiggling, participating in iterative tasks involving items like spinning wheels and lining up toys, and seeking sensory stimulation. In contrast, “Insistence on Sameness” (IS) behaviors are categorized as “higher-level” and preponderantly cognitive, marked by a strict adherence to routines, ritualistic behavioral patterns, intense and restricted interests, and a strong resistance to change (10). The differentiation is critical because these subtypes seem to follow distinct developmental paths, possess varying neurobiological foundations, and exhibit diverse connections with cognitive functions.

Recognizing ASD as a lifelong condition underscores the importance of comprehending the evolution of RRBs throughout the lifespan to offer appropriate support and intervention at different life stages. The presentation and consequences of RRBs are not fixed; they undergo changes from infancy through childhood, adolescence, and into adulthood (11, 12). The approach employed in this review, adopting a life course perspective, entails using the individual’s complete developmental trajectory from birth to adulthood as a framework to dynamically track the occurrence, evolution, and outcomes of developmental and behavioral phenotypes. The goal is to offer accurate and useful clinical references for professionals in the field, aiding in the development of a continuous support system for “early identification - standardized assessment - individualized intervention,” ultimately enhancing the identification and intervention efficacy of RRBs and supporting long-term well-being and quality of life for autistic individuals and their families.

## The heterogeneity and classification of RRBs

The range of behaviors within the RRB domain in ASD is extensive, encompassing variations in form, frequency, and purpose. To address this diversity, researchers have devised classification systems and evaluation instruments. The DSM-5 outlines four categories of RRBs (1): stereotyped or repetitive motor movements, use of objects, or speech (2); insistence on sameness, inflexible adherence to routines, or ritualized patterns of behavior (3); abnormal intensity and fixation on highly restricted interests; and (4) hyper- or hyporeactivity to sensory input or unusual interest in sensory aspects of the environment (13).

Empirical studies, primarily employing factor analysis of standardized assessment instruments, consistently uphold a two-factor configuration of RRBs, differentiating between “Repetitive Sensorimotor” (RSM) behaviors and “Insistence on Sameness” (IS) behaviors (14). A recent study validated this structure by identifying factors such as “Repetitive Sensorimotor” behaviors (e.g., hand/finger mannerisms) and “Insistence on Sameness” behaviors (e.g., resistance to change) in a substantial clinical sample of individuals with ASD (15).

The DSM-5 four-domain description and the empirically derived two-factor model are best understood as complementary rather than competing frameworks, although they are not interchangeable. The DSM-5 domains are descriptive, clinically oriented criteria intended to support reliable diagnosis across heterogeneous presentations, whereas the RSM and IS factors were derived from factor-analytic studies of standardized instruments and appear to represent dimensions with partly distinct developmental courses and correlates (14, 15). Mapping between the two frameworks is only approximate: DSM-5 domain 1 (stereotyped or repetitive motor movements, use of objects, or speech) corresponds most closely to RSM, whereas domains 2 (insistence on sameness) and 3 (highly restricted interests) load predominantly on IS. Domain 4 (hyper- or hyporeactivity to sensory input) does not map cleanly onto either factor and appears to be cross-cutting, having been related to both RSM and IS in some studies (16, 17). Consequently, the two-factor model is most useful for research that aims to resolve mechanistic or developmental heterogeneity, whereas the DSM-5 domains remain the basis for clinical diagnosis; neither framework should be assumed to supersede the other.

It is also important to recognize that not all RRBs are equally specific to autism. Several RRB forms occur, at lower intensity or frequency, in typically developing children, particularly early repetitive movements and routines that peak in the second and third years of life and subsequently decline (18, 19). What tends to distinguish autistic children from typically developing children is therefore less the mere presence of repetitive behavior than its pattern: autistic children show a broader range and greater intensity of RRBs, more persistent motor stereotypies beyond the preschool years, and more frequent atypical sensory behaviors (20, 21). Prospective and longitudinal studies indicate that elevated and persistent RRBs—especially sensory-related and circumscribed-interest behaviors—are among the more discriminating early features, although individual RRBs are not diagnostic in themselves and their specificity varies with age and developmental level (20–22).

Both the Autism Diagnostic Interview-Revised (ADI-R) and the Autism Diagnostic Observation Schedule (ADOS) are established as gold-standard diagnostic measures, encompassing segments designed for the assessment of Restricted and Repetitive Behaviors (RRBs) (5). For more detailed characterization, the Repetitive Behavior Scale-Revised (RBS-R) is a widely used caregiver scale that captures the breadth of RRBs (23). During factor analysis of the RBS-R, a five-factor solution was determined, encompassing “Stereotypic Behavior,” “Self-injurious Behavior,” “Compulsive Behavior,” “Ritualistic/Sameness Behavior,” and “Restricted Interests (23). These distinct factors emphasize the multidimensional nature of RRBs and stress the importance of evaluating specific subtypes rather than a single, aggregated score.

The regulation of RRB expression is significantly influenced by individual elements including cognitive ability, language skills, age, and sex. Lower nonverbal intelligence correlates with increased severity of RSM behaviors during early childhood and a diminished rate of progress over time. In contrast, behaviors associated with IS appear to have a weaker correlation with nonverbal IQ but may be more pronounced in individuals with milder social-communicative impairments (14) (**Figure 1**). Sex-based differences are also emerging as an important aspect of RRB heterogeneity. In individuals with ASD, females might display RRBs of lower severity and frequency than their male counterparts, both generally and with regard to specific symptoms, according to certain studies (24). Such apparent sex/gender differences should nevertheless be interpreted cautiously, because they may be influenced by ascertainment and referral biases, diagnostic practices, differences in phenotype and co-occurring conditions, age at assessment, and sampling strategy, and reported male–female differences in RRBs are not consistent across studies (22, 24). These findings highlight the need to consider individual characteristics when assessing and classifying RRBs.

**Figure 1:**
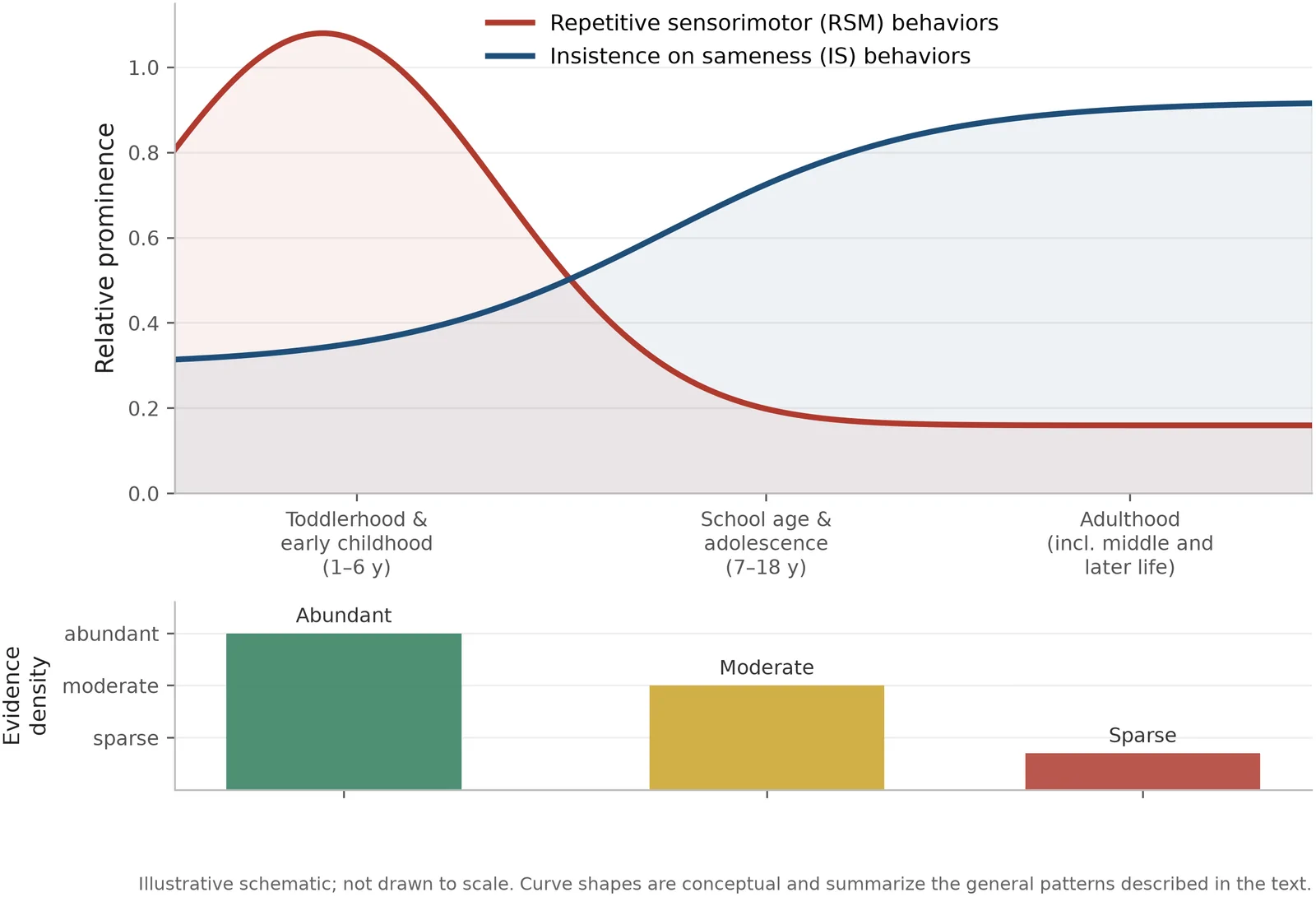
Schematic representation of the developmental trajectories of RRB subtypes across the lifespan. Repetitive sensorimotor (RSM) behaviors are typically most prominent in toddlerhood and early childhood and tend to decline with age, whereas insistence on sameness (IS) behaviors often become relatively more prominent during school age and adolescence and remain present into adulthood. The panel above indicates the relative density of available evidence across life stages: evidence is most abundant in early childhood, moderate in school-aged children and adolescents, and sparse in middle-aged and older autistic adults. The schematic is illustrative and is not drawn to scale.

## Assessment and measurement of RRBs across the lifespan

Conclusions about developmental change in RRBs depend heavily on the instruments used, because different measures target different RRB subtypes, rely on different informants, and are calibrated to different ages (23, 25). For more detailed characterization, caregiver-report instruments such as the RBS-R and the Repetitive Behavior Questionnaire-2 (RBQ-2) capture the breadth of RRBs in young children, whereas adult versions—the RBQ-2A and the Repetitive Behaviors Questionnaire-3 (RBQ-3)—allow self-report in adolescents and adults, and the RBQ-3 additionally permits informant report (23, 26–28).

Several practical properties are important when selecting a measure in a lifespan context. With respect to age appropriateness, some instruments are validated only within narrow age bands (for example, the RBQ-2 for two-year-olds and the RBS-R in young children), whereas others have been extended to adolescents and adults (26, 29, 30). With respect to informant, most child measures rely on caregiver report, which may under-detect internal states and subtle behaviors, whereas adult measures increasingly use self-report; agreement between self- and informant reports is moderate at best, and each perspective captures partly different information (29, 31). With respect to sensitivity to change, many RRB instruments were developed for descriptive or diagnostic purposes and were not designed as treatment endpoints; where they have been evaluated in this role, their responsiveness has often been modest or insufficiently established (25). Taken together, these limitations mean that apparent developmental differences in RRBs may partly reflect differences in the measures used at different ages, and cross-age comparisons should be made with caution. **Table 1** summarizes commonly used measures and their principal properties.

**Table 1:** Selected measures of restricted and repetitive behaviors and their principal properties across the lifespan.

Instrument	RRB coverage	Informant/method	Typical age range	Sensitivity to change and suitability
ADI-R	RRB subdomain (lifetime presence/absence)	Caregiver interview (clinician-administered)	From ~2 years (mental age ≥2 years) onward	Designed for diagnosis; RRB section is brief and not optimized for detecting change over time (5, 31).
ADOS-2	RRB items within a standardized observation	Direct observation by trained examiner	Toddlerhood to adulthood (module chosen by age/language)	Standardized observation; limited RRB item coverage; less suited to repeated measurement (31).
RBS-R	Five factors (stereotypic, self-injurious, compulsive, ritualistic/sameness, restricted interests)	Caregiver report	Validated from young children (~2–3 years) to adults	Broad coverage; moderate length; responsiveness as a change measure not fully established (23, 29).
RBQ-2	Repetitive and restricted behaviors in early childhood	Caregiver report	infants ≥15 months of age	Age-specific and useful in toddlerhood; not applicable across the lifespan (18, 19).
RBQ-2A	RSM and IS subscales	Self-report	Adolescents and adults	Extends RRB assessment to verbally fluent adults; self-report only (27, 28).
RBQ-3	RSM and IS subscales	Self-report and informant report	Toddlerhood to adulthood	Allows both self- and informant perspectives; recently psychometrically evaluated (29).
Subcategory/factor measures (e.g., RSM vs IS)	RSM vs IS dimension scores	Derived from the instruments above	Children (mostly)	Useful for subtype-specific research; comparability across ages depends on the source instrument (17, 30).

Clinical and research settings should therefore select measures appropriate to the developmental stage and to the question at hand, ideally combining caregiver report, self-report where feasible, and direct observation, and should interpret change over time with explicit attention to the measurement properties of the instrument used.

## Underlying mechanisms of restricted and repetitive behaviors

The etiology of RRBs is complex, involving an interplay of genetic, neurobiological, and psychological factors (32).

### Genetic factors

ASD is highly heritable, and genetic factors contribute to RRBs; however, most genetic evidence concerns ASD as a whole rather than RRBs specifically. Large-scale genomic studies have identified numerous risk loci for ASD, a substantial proportion of which converge on neurodevelopmental processes and synaptic function (33). Quantitative-genetic analyses further suggest that the social and non-social symptom domains of autism are partially genetically dissociable, indicating that RRB-related variation may have partly distinct genetic underpinnings (34). Direct, RRB-specific human genetic evidence nevertheless remains limited, and much of the gene-level evidence summarized below derives from animal models or from monogenic syndromes in which RRBs are one feature among many rather than from studies that index RRBs directly. **Table 2** therefore presents selected illustrative examples rather than an exhaustive list, and the relationship reported for each entry should be read at the level of the phenotype studied (syndrome or animal model) rather than as a gene-level effect specific to RRBs.

**Table 2:** Selected illustrative examples of ASD-associated genes and monogenic syndromes with reported repetitive-behavior phenotypes.

Gene/syndrome	Evidence type	Reported relationship with RRBs	Reference
SHANK3 (Phelan–McDermid syndrome)	Mouse models	Mice carrying Shank3 mutations show excessive self-grooming and other stereotyped behaviors, linking a synaptic scaffolding protein to repetitive behavior.	(35, 36)
SHANK1	Mouse model	Shank1-knockout mice display repetitive behaviors (e.g., self-grooming) alongside social and cognitive alterations.	(37)
ADNP (Helsmoortel–van der Aa syndrome)	Human monogenic syndrome; mouse models	*De novo* ADNP mutations cause a syndromic form of autism (Helsmoortel–van der Aa syndrome) in which repetitive behaviors are frequently reported as one feature of a broader neurodevelopmental phenotype; this denotes a syndrome-level association rather than a direct ADNP–RRB relationship. Evidence that the ADNP-derived peptide NAP reduces repetitive behavior derives from Shank3-mutant mice and does not establish a direct ADNP–RRB link.	(38–40)
CHD8	Mouse model	CHD8 haploinsufficiency in mice produces autistic-like phenotypes including increased repetitive behaviors.	(41)
SYNGAP1	Human genetic syndrome	SYNGAP1-related intellectual disability has a behavioral phenotype that includes repetitive behaviors.	(42)

### Neurobiological basis

Differences in brain structure and function in individuals with ASD have been identified through neuroimaging studies throughout their lifespan (43). Much of this evidence derives from studies of ASD samples in general rather than from studies that isolate RRBs; where a finding relates to RRBs specifically, this is stated explicitly. Through an extensive mega-analysis carried out by the ENIGMA ASD working group, it was found that ASD is linked to decreased subcortical volumes (pallidum, putamen, amygdala) and modified cortical thickness distributions, especially in frontal and temporal regions (44). These regions are components of cortico-striato-thalamo-cortical (CSTC) loops, crucial for motor control, habit formation, and executive function, all pertinent to RRBs. Investigations in recent years have emphasized brain network dynamics, demonstrating that disrupted connectivity in the limbic circuit, particularly involving the orbitofrontal cortex (OFC) and amygdala, is linked to the severity of RRBs (45). At a molecular level, there is growing evidence that oxidative stress could play a crucial role in ASD pathophysiology. Dysregulation of the NRF2 signaling pathway may contribute to redox imbalance, impacting neurodevelopmental processes associated with RRBs (32). Additional evidence from animal models indicates that environmental enrichment can reduce restricted repetitive behavior, suggesting that experiential and environmental factors also modulate RRB expression (46).

### Psychological theories

Several psychological theories have been proposed to explain RRBs. The Executive Dysfunction Theory suggests that impairments in higher-order cognitive functions, including cognitive flexibility (shifting), inhibitory control, and planning, contribute to the perseverative and inflexible nature of RRBs (45). Supporting this, one study found that better inhibitory control and cognitive flexibility were specifically related to fewer higher-order RRBs (e.g., restricted interests, repetitive routines) in school-aged children with ASD (47). A recent meta-analytic synthesis similarly reported consistent, albeit moderate, associations between executive function skills and RRBs, while emphasizing that the strength of these associations varies by RRB subtype and by the executive function domain examined (48).

One other prominent theory suggests that RRBs serve to regulate anxiety and arousal levels in individuals (6). The predictability and rhythmicity of repetitive behaviors may induce a calming impact on a sensory environment that is overwhelming or unpredictable, aligning with the elevated prevalence of co-occurring emotional distress in individuals with ASD (5) and the observation that RRBs often increase during times of stress. The role of anxiety in driving RRBs may represent a crucial differentiator between ASD and other conditions characterized by repetitive behaviors, like obsessive-compulsive disorder (OCD) (49, 50).

Beyond anxiety and arousal regulation, several additional functions of RRBs have been proposed. These include sensory regulation (modulating the intensity of sensory input), the maintenance of predictability and control over an environment, emotional regulation, the experience of enjoyment and interest, and the construction of a coherent sense of self and identity, particularly through intense circumscribed interests (51). Repetitive behavior may also help autistic individuals make sense of, and exert some control over, an environment experienced as unpredictable or overwhelming (46). These functions are not mutually exclusive and may differ across RRB subtypes and developmental stages; for example, insistence on sameness appears to be more closely linked to anxiety and to effortful control than repetitive sensorimotor behavior (51).

## Developmental trajectories of RRBs across the lifespan

The manifestation of RRBs is not fixed but undergoes dynamic changes throughout an individual’s lifespan. It is essential to comprehend these developmental paths for precise diagnosis, prognosis, and determining the appropriate timing for interventions.

### RRBs in toddlerhood and early childhood (ages 1-6)

RRBs represent a fundamental characteristic evident at an early stage of development and contribute to the prompt identification of ASD (52). It should be kept in mind, however, that repetitive behaviors are also common in typically developing toddlers, in whom they generally peak in the second and third years of life and then decline; in autism, they tend to be more frequent, more varied, and more persistent (18, 19). In toddlers and young children, RRBs are often dominated by lower-level motor and sensory behaviors. In a study involving toddlers with ASD, sensory-related RRBs were identified as the most prevalent type, followed by circumscribed interests and object stereotypies (53). Repetitive body movements like hand-flapping or toe-walking, intense visual inspection of items, and atypical sensory interests are common manifestations. During this period, restrictive behaviors also manifest prominently in feeding and eating habits. Food selectivity, distinguished by a narrow scope of acceptable dietary choices, frequently linked to sensory attributes such as tactile qualities or color, is notably more prevalent among children with ASD compared to their typically developing counterparts (54). This behavior can be conceptualized as an RRB, evidencing a commitment to uniformity and heightened sensory sensitivities (55). These eating difficulties are often linked to gastrointestinal (GI) problems, which are also more prevalent in autistic children (56, 57). Several studies suggest that these dietary choices could influence the link between ASD and gut microbiome alterations, advising against asserting a direct causal role of the microbiome in the core characteristics of ASD (58).

### RRBs in school-aged children and adolescents (ages 7-18)

As children with ASD move into school age and adolescence, the pattern of RRBs continues to evolve. Generally, there is a trend for RRBs to become less frequent and severe with increasing age (12). While lower-level motor stereotypies may decrease or become more subtle, higher-level RRBs, such as ritualistic actions and circumscribed interests, tend to become more prominent (59). Circumscribed interests often become more complex and abstract, focusing on specific topics like train schedules, historical events, or scientific facts. These interests can provide enjoyment and expertise, yet they might disrupt social interactions and academic responsibilities if they overshadow conversations and activities.

During this developmental stage, the association between RRBs and co-occurring mental health challenges becomes more apparent. Research has shown that specific RRB subtypes, particularly “Ritualistic/Sameness” behaviors, are significant predictors of both internalizing problems (e.g., anxiety) and externalizing problems (e.g., aggression) after controlling for age and cognitive level (59). The necessity for predictability and consistency can result in significant anxiety, and deviations from these routines can incite distress or challenging behaviors. The existence of RRBs, coupled with social challenges, can elevate the risk of being bullied and victimized by peers (60).

### RRBs in adulthood

Research on RRBs in autistic adults, especially in middle and late life, is significantly constrained (11, 61). The evidence base for middle-aged and older autistic adults is particularly thin, and the conclusions below therefore refer mainly to younger autistic adults; findings should not be assumed to generalize to later life. The existing evidence indicates that although the fundamental inclination for RRBs remains, their manifestation and purpose can evolve. Certain obvious motor stereotypies might diminish or transform into subtler or socially sanctioned actions. Limited interests frequently persist, posing both remarkable talents and notable social obstacles.

In a systematic review of self-reports by autistic adults, the sensory aspect of RRBs emerged as a central theme, with numerous adults reporting hypersensitivity to particular stimuli (62). These adults often use repetitive actions as a form of self-regulation to cope with stress and sensory overstimulation. This underscores the adaptive function that RRBs can fulfill, a viewpoint essential for formulating interventions that are supportive rather than solely suppressive. As the autistic population ages, it is crucial to contemplate the potential interplay between age-associated changes in brain health, including declines in glymphatic and vascular integrity, and the neurodevelopmental profile of ASD, which may impact the manifestation of RRB (63).

## Cultural and contextual influences on RRBs

Cultural norms and diagnostic practices shape whether a given behavior is perceived as problematic, adaptive, or in need of intervention, and they also influence how caregivers report and prioritize RRBs (64, 65). For example, the acceptability of intense circumscribed interests or of particular routines may vary across cultural contexts, and the threshold at which a behavior is judged to warrant clinical attention is not culture-free (65). Cross-cultural validation studies indicate that RRB instruments developed in high-income, predominantly Western settings do not always behave identically when translated and applied elsewhere: although the RBS-R has shown broadly acceptable psychometric properties in Chinese children aged 3–8 years, and RRB profiles and correlates have been described in other non-Western samples, factor structures and item functioning can differ across populations (66). Evidence of diagnostic disparities and delayed identification among racial, ethnic, and Indigenous minority groups further indicates that cultural and systemic factors influence access to assessment and the interpretation of RRB-related concerns (65, 67, 68). These considerations reinforce the need to interpret RRB findings with attention to the cultural and service context in which they were obtained, and to exercise caution when generalizing measures and norms across populations.

## Clinical interventions for RRBs

Managing RRBs is a central goal of clinical intervention for ASD. Approaches can be broadly divided into non-pharmacological and pharmacological strategies. **Table 3** summarizes the interventions discussed in this section, together with their target symptoms, evidence base, and level of evidence maturity. As noted above, a recurring limitation of this literature is that many studies were conducted in ASD samples generally and did not isolate RRBs as the primary outcome.

**Table 3:** Summary of interventions for RRBs: target symptoms, evidence base, principal outcomes, limitations, and evidence maturity.

Intervention	Target symptom/RRB subtype	Study design/evidence level	Population	Principal outcome	Key limitations	Evidence maturity
Behavioral interventions (ABA-based/function-based)	Specific, operationally defined RRBs	Single-case experimental designs; small trials; reviews	Children (mostly)	Reductions in targeted behaviors; evidence for core RRB reduction remains limited	Heterogeneous designs; small samples; variability in outcome measures	Established for targeted behaviors (69, 70)
Risperidone/aripiprazole	Irritability, aggression, self-injury (associated symptoms)	RCTs; network meta-analysis	Children and adolescents	Reduce irritability/aggression; effect on core RRBs unclear	small samples, considerable heterogeneity exists among studies.	Established for associated Symptoms (71, 72)
Acetylcholinesterase inhibitors	Cognition/attention; core ASD features	RCTs; Cochrane review	Children	No established benefit for core features, including RRBs	A high risk of bias and serious uncertainty remain.	Investigation al — not supported (73)
Memantine	Core ASD symptoms	RCTs	Children and adolescents	Lack of evidence of benefit	Negative	Investigation al — not supported (74)
Fecal microbiota transplantation (FMT)	GI symptoms; ASD-related behaviors	Open-label study	Children	Improvements in GI and behavioral measures	No blinding or control; no RRB-specific endpoint	Preliminary (75)
Probiotics (L. reuteri)	Social function	Double-blind RCT	Children	Improved social behavior; no effect on RRBs	No RRB benefit;	Preliminary (76)
Cannabinoids (CBD-rich extract)	Hyperactivity, irritability, psychomotor agitation	RCT; systematic review	Children	Some improvement in psychomotor agitation	Small and short-term; long-term safety unknown	Preliminary (77, 78)
Non-invasive brain stimulation (tDCS/rTMS)	Core ASD features, including RRBs	Network meta-analysis of RCTs	Mixed ages	No significant improvement in RRBs	Heterogeneity; short follow-up	Investigational (79)
Deep brain stimulation (DBS)	Severe, treatment-resistant self-injury	Case series/small studies	Adolescents and adults	Possible benefit in selected severe cases	Invasive; very limited evidence	Preliminary (80)
NRF2-targeted antioxidants	ASD-related behaviors, including RRBs	randomized, double-blind, placebo-controlled clinical trials	Children and adolescents	Mechanistic rationale; clinical evidence lacking	small samples	Preclinical (32)
Digital motor markers	Core ASD features	Developmental/feasibility studies	Mixed ages	Potential for earlier detection	small samples; standardized data are lacking.	Preclinical/emerging (81)

### Non-pharmacological interventions

Behavioral interventions, like those grounded in the principles of Applied Behavior Analysis (ABA), are widely regarded as the primary and most efficacious intervention for addressing the core deficits of ASD, encompassing RRBs (69). In most cases, these treatments necessitate a functional behavior assessment to ascertain the antecedents and reinforcers underlying a specific RRB. Based on this analysis, strategies are devised to educate on alternative, adaptive behaviors and adjust the environment to decrease triggers for the RRB. Evidence for behavioral interventions is strongest for reducing specific, operationally defined repetitive behaviors in single-case experimental designs and small trials, whereas evidence from large randomized controlled trials targeting core RRBs as a primary outcome remains limited, and the quality of evidence varies with the behavior targeted and the outcome measure used (70). Despite the efficacy documented by behavioral methods, there is an acknowledged requirement for more systematic and targeted approaches customized to the specific subtype and function of the RRB (6).

Intervention is generally indicated when a behavior is harmful or interferes with functioning or quality of life(for example, self-injury or behaviors that obstruct learning), whereas behaviors that are benign or that serve a valued function for the individual (for example, competence, and expertise) may not require reduction and may instead warrant accommodation or support (82). Concerns have also been raised that some behavioral approaches have historically prioritized normalization and compliance over the individual’s well-being, underscoring the importance of selecting goals in collaboration with the autistic person and their family and of attending to the autistic community’s perspectives on intervention (82, 83).

### Pharmacological interventions

While no medication is approved for core ASD symptoms, several are prescribed to manage related challenging behaviors. Atypical antipsychotics, like risperidone and aripiprazole, are extensively studied and frequently employed in managing associated behaviors such as severe irritability, aggression, and self-injury, commonly linked to RRBs (71). Despite this, the impact on the core RRBs remains unclear, with a risk of notable side effects, including weight gain. This distinction—between effects on associated symptoms and effects on core RRBs—should be maintained consistently: available medications are directed at associated symptoms rather than at RRBs themselves.

Additional medications have been studied, with outcomes that are inconclusive. Acetylcholinesterase inhibitors, utilized in dementia management, have been explored for their potential to enhance cognitive functions like attention. Nevertheless, their influence on core autism features, including RRBs, has not been definitively established (73). In a similar vein, a systematic review of memantine, another dementia medication, revealed a lack of evidence supporting its effectiveness in reducing the core symptoms of autism (74). Consistently, a systematic review and meta-analysis of pharmacotherapy for restricted/repetitive behavior concluded that the evidence that available medications reduce core RRBs is limited and inconclusive, and that current pharmacological treatment is directed mainly at associated symptoms rather than at RRBs themselves (72).

### Novel and emerging interventions

Research is rapidly expanding to explore novel therapeutic avenues, highlighting the pressing demand for enhanced and precise treatments. The interventions grouped in this section differ markedly in their evidence maturity and should not be regarded as equivalent. We therefore distinguish between: (i) interventions that are established or clinically used for associated symptoms (for example, risperidone and aripiprazole, discussed below); (ii) investigational interventions that have been evaluated in controlled human studies with mixed or limited results (for example, gut-microbiota–targeted approaches and non-invasive brain stimulation); (iii) preliminary interventions supported mainly by small, open-label, or single-arm studies (for example, cannabidiol-rich cannabis extracts); and (iv) preclinical or proof-of-concept approaches supported largely by animal or mechanistic work (for example, NRF2-targeted antioxidants and digital motor markers as early-detection tools). None of the approaches in categories (ii)–(iv) has yet been shown to produce robust, replicated improvements in core RRBs, and their therapeutic potential should not be overstated (46, 72).

Due to the elevated frequency of gastrointestinal issues in individuals with ASD, the “microbiome-gut-brain axis” has emerged as a key area of research (59). Several studies have investigated the manipulation of the gut microbiota to enhance symptoms associated with ASD. An open-label study on fecal microbiota transplantation (FMT) reported improvements in both GI and ASD symptoms, including behavioral issues (75). Probiotics were assessed in a randomized controlled trial, where a combination of L. reuteri strains was shown to enhance social functioning in children with ASD. Nevertheless, there was no significant effect on RRBs or the overall severity of autism (76).

The therapeutic potential of cannabis-derived products is increasingly attracting attention. A systematic review revealed that cannabis and cannabinoids could alleviate various ASD-related features, including hyperactivity, irritability, and psychomotor agitation, commonly linked to RRBs (77). Children with ASD demonstrated notable enhancements in psychomotor agitation when given a CBD-rich cannabis extract in a randomized, placebo-controlled trial (78). However, more investigation is needed to establish long-term safety and effectiveness.

Brain stimulation techniques aim to modulate the activity of neural circuits directly implicated in ASD. In a network meta-analysis of non-invasive brain stimulation (NIBS) treatments, including transcranial direct current stimulation (tDCS) and repetitive transcranial magnetic stimulation (rTMS), it was determined that none of these treatments significantly improved RRBs in individuals with ASD (79). In cases of severe, treatment-resistant conditions, especially those with life-threatening self-harm, deep brain stimulation (DBS) has been investigated as a potential, albeit invasive, intervention (80).

The discovery of pathways such as NRF2-mediated oxidative stress at the molecular level has paved the way for investigating specific treatments. Currently, antioxidant compounds that aim to reinstate NRF2 function are being studied as a potential strategy to alleviate ASD-related behaviors, particularly RRBs (32). Furthermore, progress in technology, including employing digital motor markers for early detection, holds promise for facilitating earlier and more timely treatments, which have been shown to yield superior long-term results (81).

Current diagnostic tools tend to emphasize the social and behavioral deficits of autistic children, rather than highlighting their cognitive and perception-based abilities and strengths. This framing carries broad implications for potential interventions and behavioral therapeutic strategies. Numerous contemporary therapeutic strategies for individuals with autism concentrate exclusively on addressing deficiencies in social skills and aim to improve these impairments through direct exposure. Although these therapeutic interventions are expected to benefit numerous individuals in achieving a satisfying life and enhancing adaptive functioning, fostering self-esteem and promoting positive mental health in autistic children may lead to enhanced quality of life by equally emphasizing their cognitive and perception-based strengths and interests. Strength-informed intervention, as an alternative strategy, emphasizes leveraging autistic interests and abilities observed in natural environments, typically manifested through repetitive behaviors and engagement with tangible items (82).

## Discussion

This review highlights that restricted and repetitive behaviors (RRBs) in ASD are a multifaceted, varied, and enduring characteristic. The developmental trajectory of RRBs shows a general shift from predominantly sensory-motor manifestations during early development to more cognitively driven RRBs in adolescence and adulthood. This trajectory, however, is not uniform, and it is important to state that the strength of the underlying evidence differs markedly between life stages. The evolution highlights the importance of incorporating a lifespan perspective in clinical assessment and research, shifting from cross-sectional investigations to longitudinal designs that can effectively capture these dynamic changes.

The diversity of RRBs presents a significant challenge, as grouping varied features together may mask crucial distinctions in their fundamental mechanisms, purposes, and reactions to treatments. Future research should focus on deconstructing the RRB domain into more refined, phenotypically homogeneous subgroups (84). This would assist in pinpointing specific biomarkers and in creating more targeted interventions.

A critical aspect often overlooked in intervention research is the function of RRBs for the autistic individual. These behaviors, although potentially impairing, can also provide comfort, predictability, and pleasure, serving as effective coping mechanisms for anxiety and sensory overload (6, 62, 84). Thus, interventions should not always aim for behavior elimination. For a more nuanced approach, consider the influence of the behavior on the individual’s quality of life, and ascertain whether the priority should be to decrease, replace, or adjust it (82). For example, a specific interest could be utilized in educational and professional environments to enhance learning and job opportunities.

The current landscape of interventions for RRBs reveals a significant gap. Behavioral therapies require significant time and effort and may not be accessible to all individuals. Pharmacological options mainly focus on addressing associated symptoms such as irritability rather than directly targeting the core RRBs, often accompanied by significant side effect profiles (71). Novel treatments like neuromodulation and microbiome-based therapies are promising but remain at the preliminary stages of investigation, with limited studies for their effectiveness on RRBs specifically (76, 79). Bridging the gap between our growing understanding of the neurobiology of RRBs and the development of mechanism-based treatments is a crucial priority for the field.

### Limitations of this review

This review has several limitations that should be considered when interpreting its conclusions. First, as a narrative review, it does not provide a systematic or exhaustive account of the literature, and the studies discussed were selected to illustrate key themes rather than to represent the full evidence base; no formal quality appraisal or certainty-of-evidence grading was performed. Second, the evidence is unevenly distributed across the lifespan: RRBs are comparatively well characterized in early childhood and reasonably studied in school-aged children and adolescents, but evidence in middle-aged and older autistic adults is sparse, and the available measures do not span the entire lifespan in a comparable way (11, 30, 61). Third, a considerable proportion of the mechanistic and intervention evidence derives from studies of ASD samples in general rather than from RRB-specific studies, and much of the gene-level evidence is derived from animal models or from monogenic syndromes. Fourth, RRB expression and its interpretation are sensitive to cultural and service context, so findings obtained in one population may not generalize directly to others (65). These limitations should be borne in mind when applying the findings of this review to individual clinical decisions.

### Future directions

Future work should prioritize longitudinal studies that extend from childhood into adulthood and later life, so that developmental trajectories can be described with measures that are comparable across ages (30). In addition, research should increasingly be conducted in partnership with autistic individuals. Participatory and co-produced approaches, in which autistic people help to define research questions, interpret findings, and set priorities, are particularly valuable for a construct such as RRBs, where assumptions about what counts as a problem may not align with the lived experience of autistic people. Qualitative and participatory studies can challenge prevailing assumptions about RRBs and clarify how autistic individuals wish to be supported in managing—or indeed retaining—these behaviors (85, 86). Finally, intervention studies should routinely specify the RRB subtype targeted, use measures validated for detecting change, and report functional outcomes alongside symptom measures.

## Conclusion

In Autism Spectrum Disorder, restricted and repetitive behaviors constitute a core element, marked by intricate and evolving features over the lifespan. Significant progress has been achieved in categorizing RRBs into distinct subtypes, mapping their developmental paths from early childhood to adolescence, and initiating the exploration of their neurobiological and psychological foundations. The progress has verified that RRBs do not constitute a uniform entity; instead, they represent a diverse range of features with different developmental paths and links to cognitive function. It should be emphasized, however, that these advances are not uniform across the lifespan: the evidence base is considerably stronger for childhood than for adulthood, and conclusions about middle-aged and older autistic adults remain tentative.

Progress in the future is contingent upon a multi-pronged strategy. Intervention research must shift toward an individualized, needs-led framework in which supports are designed and evaluated according to the specific type and function of an individual’s RRBs, the degree of functional impairment or benefit they entail, and the person’s own priorities and quality of life. In addition, the field should move toward research conducted in partnership with autistic individuals, and should remain explicit about the strength and the gaps in the available evidence, particularly for later life.
